# Navigating uncharted territory with a borrowed map: lessons from setting up the BATH-OUT-2 randomised controlled trial in adult social care and housing services in English local authorities

**DOI:** 10.1186/s13063-024-08073-1

**Published:** 2024-03-25

**Authors:** Jennifer McAnuff, Tim Rapley, Leigh Rooney, Phillip Whitehead

**Affiliations:** 1 Department of Social Work, Education and Community Wellbeing, Northumbria University, Newcastle Upon Tyne, NE7 7XA UK; 2 Population Health Sciences Institute, Newcastle University, Newcastle Upon Tyne, NE4 6BE UK

**Keywords:** Social care, Housing, Randomised controlled trials, Home environment, Housing adaptations, Healthy ageing, Occupational therapy

## Abstract

Populations around the world are rapidly ageing and more people are living with multiple long-term conditions. There is an urgent need for evidence about high quality, cost-effective, and integrated systems of health and social care. Health research funders are now also prioritising research in adult social care and wider local authority settings, e.g. housing services.

Developing the evidence base for adult social care should include implementing randomised controlled trials, where appropriate. Within the UK, the clinical trial is the established road map for evaluating interventions in the National Health Service (NHS). However, adult social care and local authorities are relatively uncharted territory for trials. BATH-OUT-2 is one of the first clinical trials currently underway within adult social care and housing adaptations services in six English local authorities. It provides an opportunity to explore how the clinical trial road map fares in these settings.

Whilst setting up BATH-OUT-2, we encountered challenges with securing funding for the trial, lack of non-NHS intervention costs, using research and support costs as intended, gaining approvals, identifying additional trial sites, and including people who lack the mental capacity to provide informed consent. Overall, our experience has been like navigating uncharted territory with a borrowed map. In the UK, the clinical trial road map was developed for medical settings. Its key features are integrated within the NHS landscape but have been largely absent, unfamiliar, inaccessible, or irrelevant in social care and wider local authority terrain. Navigating the set-up of a clinical trial outside the NHS has been a complicated and disorientating journey.

BATH-OUT-2 highlights how local authorities generally and adult social care specifically are a relatively new and certainly different type of setting for trials. Whilst this poses a challenge for conducting trials, it also presents an opportunity to question longstanding assumptions within trials practices, reimagine the conventional clinical trial road map, and take it in new directions. As the UK research landscape moves forward and becomes better primed for randomised evaluations in local authorities, we propose several suggestions for building on recent progress and advancing trials within adult social care and across health and care systems.

## Background

Populations around the world are rapidly ageing and the number of people living with multiple long-term conditions is growing, resulting in major implications for health and social care systems worldwide [1–4]. There is an urgent need for evidence about high-quality, cost-effective, and integrated health and social care systems that promote health and quality of life, reduce health inequalities, focus on prevention, and tackle the wider determinants of health and wellbeing. In this context, major research funders such as the United Kingdom (UK) National Institute for Health and Care Research (NIHR) are now prioritising research in adult social care and local authorities as well as within health services [4, 5]. Similarly, the UK National Institute for Health and Care Excellence (NICE), a world leader in developing guidelines about health care, is now also committed to providing guidance for social care and local authority settings [6].

Improving the evidence base for adult social care needs to incorporate rigorous evaluation of interventions and service delivery models, including randomised controlled trials where these are useful and feasible [2, 7–9]. BATH-OUT-2 is one example of a randomised controlled trial currently underway in adult social care and housing adaptations services within six local authorities in England [10, 11]. The trial aims to determine the clinical and cost-effectiveness of bathing adaptations, specifically a level-access walk-in shower, for older people who have difficulty accessing their bath. The primary outcome is physical wellbeing and secondary outcomes include mental wellbeing, self-reported falls, perceived risk of falling, independence in daily activities, independence in bathing, perceived difficulty in bathing, and health- and social care-related quality of life. The trial is also investigating how waiting times for adaptations affect outcomes and health and social care resource use and will compare the cost-effectiveness of expedited versus routine provision of adaptations. BATH-OUT-2 aims to generate much-needed evidence for adult social care decision-makers and signals the wider ambition of building a more robust social care evidence base and further establishing large-scale, multi-site research in local authorities [4].

Within the UK, randomised controlled trials have primarily been used within medical settings and the clinical trial is firmly established as the road map for evaluating interventions in the National Health Service (NHS). In contrast, adult social care and local authorities are relatively uncharted territory for randomised evaluations of interventions [2, 3, 7, 8, 12], and there is some uncertainty about their appropriateness in these settings (e.g. [9, 13]). There are also significant differences between the NHS and local authorities when it comes to research generally. Local authorities have received much less investment in research resources and infrastructure and therefore do not have the research experience that can be widely found in the NHS [4, 14]. BATH-OUT-2 provides an opportunity to explore how the clinical trial road map, developed for and well-established within medical settings, fares in adult social care and local authorities.

Our aim for this commentary is twofold. First, we will describe six practical and methodological issues encountered whilst setting up BATH-OUT-2: securing funding for the trial, lack of non-NHS intervention costs, using research and support costs as intended, gaining approvals, identifying additional trial sites, and including people who lack the mental capacity to provide informed consent. Then, we will summarise how the UK research landscape is moving forward with these challenges and suggest what else could be done to help advance randomised controlled trials in adult social care and local authorities.

## Practical and methodological issues with setting up BATH-OUT-2

### Funding for the trial

We found it challenging to fit the BATH-OUT-2 proposal into one of the main UK funding programmes for clinical trials in the NHS. We have a conventional primary research question—the effectiveness and cost-effectiveness of intervention versus no intervention—and we built our argument for trial funding in the usual way. Previous systematic reviews had indicated the potential benefits of housing adaptations but the need for more robust evidence and the earlier BATH-OUT feasibility study had suggested that a trial using routine waiting times to form a waiting list control group would be feasible and acceptable [15–17]. However, when it came to funding for a definitive clinical trial, the non-NHS setting for BATH-OUT-2 seemed to preclude a health technology assessment and an application to a commissioned research call about age-friendly environmental interventions was unsuccessful as BATH-OUT-2 did not fit the public health brief for that funding programme [18]. This meant that, although housing adaptations are directly targeting individual and population health outcomes, BATH-OUT-2 did not seem to fit well with key funding programmes for clinical trials of health-related interventions.

### Intervention costs

Trials conducted within the NHS often involve a new intervention that costs more than standard care and may continue to be provided after the trial has been completed, thereby incurring additional ongoing costs for health service funders. The difference in cost between the new intervention and standard care is referred to as the NHS intervention cost or excess treatment cost [19, 20]. As the NHS has a statutory duty to promote research and use evidence obtained from research [21], it follows that health service funders are required to make arrangements for intervention costs. There is a national model in place in England for managing the complexities of NHS intervention costs, along with extensive local infrastructure such as agreed policies for determining the costs, processes for negotiating with health service funders, and specialist support for study teams [19, 20].

In contrast, at the time of the BATH-OUT-2 grant application and set-up, there were no funding arrangements or infrastructure in place for intervention costs in trials or other studies outside the NHS. As a workaround, some of our intervention costs were covered by the trial funder as an additional research cost. This addressed a practical problem for BATH-OUT-2 and enabled the trial to progress. However, more broadly, the lack of non-NHS intervention costs is a significant challenge to progressing intervention research in social care and local authorities [13, 22, 23]. Subsidising non-NHS intervention costs with funding protected for conducting social care research obscures the level of need for such costs and ultimately reduces the overall amount of funding available for much-needed social care intervention research, which already lags well behind the amount invested in NHS intervention research.

### Research and support costs

Research costs relate to activities carried out to answer the research question (e.g. trial co-ordination and management at sites) and support costs relate to additional patient care activities associated with the research (e.g. screening patient records for study eligibility) [20]. As with trials conducted in the NHS, the research costs in BATH-OUT-2 were met by the trial funder and the support costs were met by the NIHR Clinical Research Network (CRN), which facilitates NHS and social care research across England [24]. Our research costs went towards employing BATH-OUT-2 researchers to carry out recruitment activities such as obtaining informed consent. We asked local authority sites to arrange for their own staff to screen service users’ records for trial eligibility and obtain their consent to be contacted by BATH-OUT-2 researchers. The sites would be reimbursed by the NIHR CRN for the cost of their staff time, in the form of a support cost.

Although this type of approach is common in trials conducted in the NHS, we found it challenging to work this way within BATH-OUT-2. Previous research has already highlighted that local authorities have limited time and resources available to prioritise research (e.g. [14, 25–27]), particularly since the COVID-19 pandemic. Within BATH-OUT-2, we found that local authorities were almost invariably enthusiastic about the trial, but severe pressures with workforce capacity meant that many were unable to deploy their own staff to carry out trial support activities, even though the costs would be reimbursed. We needed to rely on luck—finding and exploiting local quirks within housing adaptations services, such as a recently retired employee willing to come back to the service or a part-time employee willing to work extra hours to support the trial (many part-time employees were already working extra hours to support pandemic recovery and therefore were not available for extra hours to support research). We also relied heavily on tenacious service managers who were committed to supporting the trial despite the significant challenges they faced with everyday service delivery.

Several housing adaptations services wanted NIHR CRN researchers, rather than their own staff, to work directly at the sites screening service users for trial eligibility and obtaining their consent to be contacted about taking part in the trial. In theory, this was possible because the NIHR CRN funds researchers who are based within NHS organisations and can directly deliver research and support activities within trials conducted in the NHS. However, to date, we have been unable to deploy CRN researchers at BATH-OUT-2 trial sites. Although willing in principle to provide support, the CRN does not usually have staff in place within local authorities. In addition, the model contract governing deployment of CRN researchers is designed for the NHS context and has limited relevance for local authorities. Whilst the contract could, in theory, be more appropriately tailored to local authorities, we found that the local authorities themselves were expected to take the lead on this, which was unrealistic given their limited research resources and infrastructure compared to the NHS. Again, we have had to rely heavily on patient and committed service managers attempting to navigate these issues within their own organisations locally, invariably for the first time, and we have had limited success to date.

### Gaining approvals

Within the UK, trials involving NHS patients and social care service users are required to apply for Health Research Authority (HRA) regulatory permissions and approvals through the Integrated Research Application System (IRAS) [28]. However, it was difficult initially to have BATH-OUT-2 categorised as a trial within IRAS. The focus of BATH-OUT-2—housing adaptations for older people and the cost-effectiveness of service delivery models across different timescales—introduced uncertainty about the study design amongst system administrators and questions about whether it was *really* a ‘trial’ or should be classified as a ‘quality improvement’ or ‘implementation’ study. Once BATH-OUT-2 was established as a trial, we expected that it would undergo the standard approvals process for all clinical trials, involving an independent review from a research ethics committee (REC) and an assessment of governance and legal compliance undertaken centrally by dedicated HRA staff. However, we found that the regulatory process for trials outside the NHS was much less centralised. As BATH-OUT-2 involves adult social care service users, we knew it would be reviewed by a HRA REC authorised to review research in social care [29]. But no central assessment of governance and legal compliance was required. This struck us as a significant gap compared to the rigour with which trials within the NHS are scrutinised.

The absence of the standard central HRA checks of governance and legal compliance removed a process which can, at times, seem overly bureaucratic. However, it complicated the process of gaining approvals for the trial from individual local authorities. For multi-site trials within the NHS, the centralised HRA process aims to replace the need for individual NHS organisations to conduct their own checks and make it easier and quicker for each organisation to approve the study locally. This mechanism is not available for trials outside the NHS, and so we needed to negotiate approvals with each local authority separately. Gaining the individual approvals was challenging and time-consuming because research governance infrastructure and decision-making processes are significantly under-developed in local authorities compared to NHS organisations and there was considerable variation in process between different local authorities [14, 30–32].

Challenges in gaining approvals were further compounded by issues with research contracting. The model Non-Commercial Agreement (mNCA) [33], used commonly as the research contract for trials within the NHS and designed to also meet the requirements of non-NHS organisations, was rejected as not fit for purpose by some of our local authority sites. In all cases, it needed to be significantly changed and more appropriately tailored. For example, the mNCA language was perceived as highly medical and irrelevant, the contract did not stand up to scrutiny from local authority legal departments, and it did not sufficiently address issues of primary importance within social care, such as safeguarding and the compatibility of the research with local authority legislative duties. In combination, the issues with research contracting, the lack of a centralised approvals system, and the service delivery pressures within the local authorities meant that it usually took several months to progress from a service agreeing in principle to join the trial to being formally set up as a site implementing the study protocol. Once again, we have had to rely heavily on housing adaptations service managers driving through local authority approvals without an established process in place, usually as their first experience of taking part in research, whilst also managing services under severe pressure.

### Identifying additional trial sites

Across our initial trial sites, recruitment rates in the internal pilot phase were slower than those in the single site feasibility study [17]. This is a common challenge for trials [12] and meant that we needed to identify additional sites to reach our recruitment target. The NIHR CRN can help streamline and support site identification by gathering expressions of interest from potential sites and hosting a central portfolio of studies that organisations can use to search for trials they are able to support [24]. This infrastructure is well-established for trials within the NHS but was not viable for BATH-OUT-2. The CRN has limited reach into social care, local authorities are not aware of the portfolio and do not have research teams scanning for opportunities to contribute to trials, and the portfolio is geared towards studies within the NHS and does not index social care research.

As an alternative, we have taken a two-pronged approach to identifying new trial sites. First, as there is a strong housing adaptations practice network across England, we have publicised the trial and encouraged expressions of interest from potential sites by working with national partners including Foundations (the national body for disabled facilities grants and home improvement agencies in England), Care and Repair England (a national charity aiming to improve housing for older people, which closed in April 2022), the Royal College of Occupational Therapists’ national network of principal occupational therapists in adult social care, the Association of Directors of Adult Social Services, and the Better Care Fund team at NHS England. Second, we have also directly cold-called almost one hundred local authority housing adaptations services to discuss the trial, a task involving hundreds of emails and telephone calls as local authorities usually have no specific point of contact for research. The lack of research infrastructure and designated research roles within local authorities made the process of identifying new sites significantly more labour intensive, time-consuming, and convoluted than it usually is for trials within the NHS. There was also an element of luck involved in getting through gatekeepers at the ‘front door’ of the local authority and finding the individuals who were both interested in the trial and had the decision-making authority to agree in principle to take part. Although someone within the local authority may have been initially interested in the trial, multiple in-depth discussions with housing adaptations service managers were required to determine whether participation was feasible.

Our two-pronged approach to identifying new trial sites generated a great deal of enthusiasm about the study from colleagues on the ground in adult social care and housing adaptations services. We carried out approximately twenty preliminary meetings with services to establish their eligibility as trial sites and multiple follow-up meetings with each eligible service to discuss the local workability of the trial protocol. However, the vast majority were unable to open as trial sites. This was because of a combination of factors, several of which we have described previously including issues with workforce capacity, non-NHS intervention costs, support costs, and research contracting. Services were concerned about fitting in research alongside their everyday service delivery. We could offer limited practical support to alleviate difficulties with internal approvals processes and we were unable to deploy researchers to directly support at the sites.

### Including people who lack the mental capacity to provide informed consent

Trials within the NHS have largely excluded people who lack the mental capacity to provide informed consent to take part in research [34–36]. In contrast, this is an important service user group included in BATH-OUT-2 for two key reasons. First, older adult recipients of bathing adaptations in adult social care are a group who will have a range of medical conditions and multi-morbidities, including a high prevalence of cognitive impairment which may affect capacity to provide informed consent (e.g. related to dementia, stroke, brain injury, neurological disease, or psychosis). We believe that social care and housing adaptations professionals would expect to see this group included to maximise the generalisability of the trial. Second, when considering mechanisms of intervention effects, there may be particular benefits of bathing adaptations for older adults with cognitive impairment who rely on support from a family caregiver. The adaptations may enable a caregiver to continue to manage caring for longer, reduce the need for formal care, or prevent or delay admission to a care home, which have important implications for health and social care resource use and quality of life. These mechanisms are being investigated as important secondary outcomes in BATH-OUT-2 because they are of significant interest to adult social care funders and may be different to the ways in which bathing adaptations benefit people with physical impairments only. BATH-OUT-2 therefore fulfils the requirement of the Mental Capacity Act (MCA) specifying that, for regulatory bodies to approve research seeking to include people who lack the capacity to provide informed consent, it must be clear that the research would be less effective if it were confined to or related only to people with capacity [37]. Had BATH-OUT-2 excluded people who lack capacity, the trial would be less generalisable and have less potential to generate knowledge about the care of people with cognitive impairment.

Including people who lack the mental capacity to provide informed consent to take part introduced issues not typically encountered in trials within the NHS, because this group are usually excluded. In practical terms, our recruitment, consent, and data collection processes have been more complex than usual because they needed to satisfy the statutory requirements of the MCA as well as Good Clinical Practice Standards for research [38]. Our researchers have needed to ensure a particularly sensitive approach by tailoring their communication, developing the skills to assess and revisit capacity at each point of contact, and taking the time to identify and engage alternative decision makers [36]. We have developed separate participant information sheets and consent forms for alternative decision makers (e.g. family members acting as personal consultees) and a more complex trial database was needed (e.g. to store multiple addresses for individual participants and their personal consultees) [36].

Beyond practical matters, the key issues we encountered converged around the primary outcome, sample size, and attrition. As well as lacking the mental capacity to provide informed consent to take part in the trial, some participants would also lack the capacity to complete the self-reported primary outcome measure (the Physical Component Summary of the Short Form 36 [39, 40]). Our options were therefore to (i) exclude participants who lacked the mental capacity to complete the primary outcome measure, (ii) select an alternative primary outcome measure, or (iii) include participants who lacked the capacity to complete the primary outcome measure, with these participants forming a sub-group completing only the important secondary outcomes and measures of health and social care resource use. We decided that it was not appropriate to exclude these participants, for the reasons previously stated. We also decided that it was not appropriate to change the primary outcome measure because it had been carefully selected for its relevance, feasibility, and responsiveness, and its use was supported by earlier qualitative work and consultation with the BATH-OUT Public Involvement Group [17]. We therefore decided that a slightly larger sample size and associated extra costs would be justified by the enhanced generalisability and value to adult social care funders of the information that would be gleaned from the secondary outcomes and measures of health and social care resource use. However, an unintended consequence was the tension this introduced throughout the early stages of the trial. There were methodological concerns that people who lack mental capacity represented a problem of ‘baked-in’ attrition because they would not be self-reporting the primary outcome. Their contribution to important secondary outcomes was overshadowed by concerns about attrition and questions were posed about whether a separate trial for people who lack mental capacity would be a more appropriate way to enact the broader ethical principle of inclusion and provide the information on the secondary outcomes and health and social care resource use.

## Moving forward with trials in adult social care

We have described six practical and methodological issues encountered whilst setting up BATH-OUT-2 in adult social care and housing adaptations services within local authorities: securing funding for the trial, lack of non-NHS intervention costs, using research and support costs as intended, gaining approvals, identifying additional trial sites, and including people who lack the mental capacity to provide informed consent. Our experience of setting up BATH-OUT-2 has been like navigating uncharted territory with a borrowed map. In the UK, the clinical trial is the road map for rigorous evaluation of health-related interventions. However, it was developed for medical settings and pertains specifically to evaluation in the NHS. Many of its key features (funding, workforce, processes, etc.) are integrated within the NHS landscape but have been largely absent, unfamiliar, inaccessible, or irrelevant in social care and local authority terrain. Overall, navigating the set-up of a clinical trial outside the NHS has been a complicated and at times disorientating journey.

Recently, the UK research landscape has been moving forward and becoming better primed for randomised evaluations in adult social care and local authorities. Regarding funding for trials, the NIHR School for Social Care Research (SSCR) is a dedicated programme that understands adult social care and local authority settings and pioneers the generation of evidence in these contexts. NIHR SSCR funded BATH-OUT-2 and has supported our efforts to strike a balance between ensuring methodological robustness and pragmatically working around the challenges we have encountered. Some of the main UK funding programmes for clinical trials are now also encouraging trials within social care, or at the interface of social care and the NHS, with commissioned calls that are more sensitive to and tailored towards social care and local authority settings (e.g.[41–43]). Regarding funding for non-NHS interventions costs, policy development is underway to address the challenges for social care intervention research, and the UK Department of Health and Social Care (DHSC) and NIHR are piloting arrangements to enable intervention research in the interim [44]. Developments are also underway related to local authority research workforce and infrastructure. Thirty UK local authorities have received substantial investment to boost their research capacity [45] and support for research delivery across England will change in April 2024 when the NIHR CRN becomes the NIHR Research Delivery Network [46]. This new network aims to actively support trials in non-NHS settings and its organisational structure seeks to align with local authority boundaries. Such developments could have a significant positive impact on the types of problems we encountered with using research and support costs, gaining approvals for trials, and identifying trial sites. Regarding the inclusion in trials of people who lack the mental capacity to provide informed consent, trials are now increasingly expected to be more generalisable to diverse populations and inclusive of underserved groups, including those with impaired mental capacity where their taking part is necessary to answer the research question [4, 36, 47, 48]. This paradigm shift is particularly significant given the service user demographics in adult social care.

From our experience of setting up BATH-OUT-2, we propose the following suggestions for building on recent progress in the UK research landscape and advancing trials in adult social care and local authorities:

- ▪ Decision-making processes about funding for trials, including topics for commissioned calls and review of researcher-led funding applications, should include people with an in-depth understanding of the practice and research context in adult social care, housing, and wider local authority settings.
- ▪ In particular, practitioner researchers can support trial funders’ understanding of the evidence needed in adult social care and local authorities and can sensitise research funders to the challenges and opportunities in trial design and delivery in these settings. For example, the BATH-OUT-2 research team includes experienced occupational therapists with academic training who know the social care and local authority terrain, speak the same language as local decision-makers, and understand service user populations sufficiently to ensure inclusion of underserved groups.
- ▪ Local authorities should be enabled to build research infrastructure that makes sense within their own organisations and avoids duplication of a *clinical* research model unlikely to be relevant outside the NHS context. Research governance and regulatory pathways need to be acceptable and accessible to the adult social care workforce. Alongside new policy for non-NHS intervention costs, adult social care research teams need practical support from specialist advisors with experience and in-depth understanding of commissioning in local authorities. The NIHR CRN central portfolio of research studies should be developed to include a valid and accessible index of social care and wider local authority research that reflects the breadth and diversity of service delivery within these types of settings and organisations.
- ▪ We were almost invariably met with enthusiasm about BATH-OUT-2 from professionals working in adult social care and housing adaptations services within local authorities. Colleagues on the ground were acutely aware that trials can generate the kind of information about effectiveness and cost-effectiveness needed to inform decision-making. Overall, the principle of random allocation was well-understood and acceptable, although thoughts about feasibility were more variable. Wider exploration of professionals’ attitudes towards trials and better understanding of their feasibility concerns would help to harness this enthusiasm, inform a clearer understanding of barriers and facilitators to trials, and generate practical ways to overcome uncertainties. As part of the BATH-OUT-2 process evaluation, we are currently conducting a national survey exploring these topics.
- ▪ Recent research has explored researchers’ experiences of the barriers and facilitators to conducting trials involving adults lacking the mental capacity to provide informed consent and identified an urgent need for greater access to training and resources, supportive interventions, and tailored guidance to build capacity in this area [36]. This resonates with our experience of BATH-OUT-2. Trials researchers should also be further enabled to engage with existing good practice guidance [49, 50], which provide a starting point for understanding and valuing the contribution that adults lacking mental capacity to consent can make to evaluations in adult social care.
- ▪ We recognise that our account describes practical and methodological issues encountered in one trial conducted solely in the UK—and specifically English—context. Comparative accounts from trials in adult social care or integrated health and social care systems internationally would also inform implementation of clinical trials outside health services and medical settings.

## Conclusions

The practical and methodological issues we encountered whilst setting up BATH-OUT-2 highlight how adult social care and local authorities are a relatively new and certainly different setting for clinical trials. There are some obvious practical differences from implementing trials in NHS settings, in that attentions of health research funders have only recently turned in earnest towards social care interventions, and therefore local authorities have received much less investment in research resources and infrastructure and do not have the research experience that can be widely found in NHS organisations. But, more broadly, adult social care and local authorities have their own distinct professional groups, norms, language, values, and politics. Ideas held in these settings about what evaluation research is, who it is for, how it should be conducted, how results can be used, and where trials-related work should feature in the day-to-day priority list are likely to be quite different from the ideas held in NHS settings. Evidence from robust randomised controlled trials may be equally valued in social care and local authorities compared to the NHS but the usual machinery of trials should not be assumed to carry the same familiarity, legitimacy, relevance, or kudos. Although these differences make adult social care and local authorities a challenging new context for implementing trials, they also present a golden opportunity to reimagine the conventional clinical trial road map and question longstanding assumptions within trials practices. Adult social care and local authority engagement in trials could create a step change in how these evaluations are conducted and used for maximum population benefit both within social care and across integrated health and care systems. Rather than simply borrowing the established NHS clinical trial road map, meaningful engagement with adult social care and local authorities could help to redraw the map and take it in new directions.

## Data Availability

Data sharing is not applicable to this article as no datasets were generated or analysed.

## Abbreviations

ADASS: Association of Directors of Adult Social Services
COVID-19: Coronavirus disease
DHSC: Department for Health and Social Care
HRA: Health Research Authority
IRAS: Integrated Research Application System
MCA: Mental Capacity Act
NHS: National Health Service
NIHR: National Institute for Health and Care Research
NIHR CRN: NIHR Clinical Research Network
RCT: Randomised controlled trial
REC: Research ethics committee
UK: United Kingdom
